# Availability restrictions and mandatory precommitment in land-based gambling: effects on online substitutes and total consumption in longitudinal sales data

**DOI:** 10.1186/s12889-024-18325-z

**Published:** 2024-03-14

**Authors:** Virve Marionneau, Jani Selin, Antti Impinen, Tomi Roukka

**Affiliations:** 1 Centre for Research on Addiction, Control, and Governance (CEACG), Faculty of Social Sciences, University of Helsinki, Unioninkatu 33, Helsinki, 00014 Finland; 2 Finnish Institute for Health and Welfare, Mannerheimintie 166, Helsinki, 00271 Finland

**Keywords:** Gambling, Substitution effects, Availability, Precommitment, COVID-19, Finland

## Abstract

**Background:**

Gambling causes important harms in societies. According to the public health approach, the most effective policies to reduce harms target full populations. Availability restrictions and mandatory precommitment are among the most effective measures. However, restrictions on the availability of some gambling products or channels may also be offset by increased consumption in other products. Substitution effects can have negative public health impacts due to differing harm potential across different gambling products. This paper uses longitudinal sales data (2019–2022) from the Finnish gambling monopoly Veikkaus. During the observation period, the availability of gambling was restricted in Finland due to subsequent waves of Covid-19-related restrictions. In addition, the gambling monopoly introduced mandatory precommitment to land-based EGMs. We focus on how these restrictive policy changes impacted the total consumption of gambling and possible substitution effects.

**Methods:**

The Finnish gambling monopoly provided weekly theoretical loss data per gambling product category and gambling channel (online, land-based) for the period of January 2019 – July 2022 based on a statutory obligation. We analysed the effects of availability restrictions and other public health measures on the consumption of different products using descriptive time series and regression analyses. We compared the sale of land-based products to online equivalents at product category level and included main policy change periods in the models.

**Results:**

Total consumption of gambling declined during 2019–2022 mainly due to reduced land-based electronic gambling machine (EGM) consumption. Declines in land-based EGM sales were not offset by online alternatives or other close substitutes in the long term. However, during the first wave of Covid-19, there was an observable substitution of land-based table games by online alternatives and land-based horse betting and possibly sports betting by online horse betting. Overall, the results also show that Covid-19 functioned as a boost to an already existing trend of increasing digitalisation of gambling.

**Conclusions:**

The study provides empirical support for the effectiveness of public health-oriented policies in reducing the total consumption of gambling. Availability restrictions and mandatory precommitment are therefore likely to also reduce the burden of harms of gambling to individuals and societies.

## Introduction

Gambling can cause severe harms, ranging from financial difficulties to relationship breakdown and even suicidality. Harms are experienced by those gambling excessively, but also by many who gamble moderately. In addition, gambling harms can impact families and communities [1–3]. The most effective regulations and interventions to reduce gambling harms target populations and products rather than individual gamblers [4, 5]. Restrictions on visibility and availability, as well as mandatory ‘responsibility measures’, such as mandatory precommitment, have been shown to reduce total consumption in gambling and associated harms [6, 7]. Availability restrictions have been particularly effective for land-based gambling products [8, 9], whereas limit-setting (mandatory or voluntary) has been more typical in online environments. Little is currently known about whether restrictions in some product categories and channels impact the consumption of others. Notably, it is possible that restrictions on land-based gambling may, in some cases, lead to a substitution by online alternatives.

Previous research into the substitution effects, or market cannibalisation, across gambling products has mainly focused on the effects of new products that are introduced in a market. A systematic review into substitution effects across gambling products showed that new products typically increase the size of the aggregate market. However, some new products, notably harmful casino-type products, can also replace existing offer [10]. Much of the evidence focuses on substitution across different land-based products. Less is known about substitution across land-based and online gambling products. Furthermore, there has been little research on substitution effects in situations where gambling availability or accessibility has been reduced rather than increased. Some evidence from Norway does suggest that a ban on electronic gambling machines (EGMs) in 2007 and subsequent mandatory precommitment in reintroduced land-based EGM gambling did not initially translate into increases in online gambling, although online gambling did increase in the longer term [8].

Substitution effects may have an impact on gambling harms. Gambling products vary depending on their harm potential. Online gambling may be connected to higher problem gambling prevalence than land-based gambling (e.g., [11, 12]). Online gambling formats incorporate many harmful characteristics, such as constant availability and accessibility, intensity, and cross-selling using player data [7, 13]. From a public health perspective, restrictions on land-based gambling should therefore not lead to equivalent increases in online alternatives.

Recently, the Covid-19 pandemic and related restrictions on land-based gambling have provided new evidence on the impacts of reduced availability. The pandemic brought along unprecedented restrictions on individual movements and consumption patterns, including closures of land-based gambling venues across jurisdictions. Emerging research into the impacts of Covid-19 on gambling suggests that the precautions taken to limit the spread of Covid-19 lessened the burden of harms caused by gambling. Research has reported reductions in total consumption as well as experienced gambling harms (e.g., [14–16]). Furthermore, reductions in land-based gambling consumption do not appear to have translated into increases in online gambling at a population level in most contexts, but shifts have occurred in some sub-groups of gamblers [14, 17–25]. In a study employing account-based data on online sports bettors before and after the first weeks of the Covid-19 pandemic from several European countries no substitution of sports betting by casino games was observed. However, there was a clear reduction in wagers on sports betting as well as on casino games [25]. A Canadian population study suggested that 17% of gamblers migrated to online gambling during the first lockdown [26]. There are also indications that online gambling has increased among those who already gambled online before the pandemic [27].

Most evidence on the impacts of Covid-19 concerns the first wave (spring 2020). The few studies focusing on longer-term effects of availability restrictions have mainly used self-reported data [28, 29]. These studies have found little overall changes in gambling behaviour and only small migration from land-based gambling to online alternatives. One study from Sweden [30] has also explored gambling consumption patterns during the prolonged Covid-19 pandemic (2019–2021) using gambling company sales data. The study found that contrary to self-reported data, gambling company tax data indicated an overall increase in gambling consumption during the pandemic. The same study found some substitution of sports betting by horse race betting during spring 2020, but no significant substitution of land-based gambling by online alternatives.

In the current study, we use longitudinal sales data (2019–2022) by gambling product categories and channels (online, land-based) handed over by the Finnish gambling monopoly Veikkaus. The aim of the study is to investigate how restrictive policy changes in land-based gambling impact total consumption (sales) by product categories as well as substitution by online alternatives. During the observation period, the Finnish land-based gambling market underwent two important types of restrictions. First, Covid-19-related policies as well as an on-going reduction of the number of non-casino EGMs restricted the physical availability of land-based gambling. Second, the Finnish gambling monopoly introduced mandatory precommitment to land-based EGMs and table games. This measure had previously only been available in online gambling. In the following, we present our data and statistical methods as well as the results on how these restrictions impacted sales of different product categories. We discuss the results in light of their implications for public health and harm prevention.

## Data and methods

### Context

Finland has had a comparatively high population-level gambling participation. According to the most recent population study, 78% of the adult population had gambled in the past 12 months [31]. Before the pandemic, in 2019, the land-based channel made up 68% of Veikkaus sales [32]. 64% of the population gambled on lotteries, 47% on scratch cards, and 31% on non-casino EGMs [31]. Land-based gambling products are sold widely in the resale network, consisting for example of supermarkets, kiosks, petrol stations, and arcades of the gambling monopoly Veikkaus.

During Covid-19, Finland did not impose strict lockdown measures. While movements in public spaces were discouraged, most gambling resale points remained open and continued to sell lottery and betting products. However, EGMs and Veikkaus arcades with table and EGM products were closed to limit the risk of infection. Qualitative evidence suggests that there may have been some reductions in purchases of other land-based products due to the risk of infection, but the closure of land-based EGMs was the most salient Covid-related restriction on land-based gambling in Finland [33]. Covid-19 also impacted gambling consumption in online environments. Initial concerns over gambler migration to the online channel led the authorities to implement lowered mandatory limits on fast-paced online gambling products (bingo, scratch cards, EGMs, and other casino-type products other than poker) during spring 2020.

Alongside Covid-19-related restrictions, the Finnish gambling field has undergone other public health-oriented reforms in recent years. Starting in January 2020, the number of land-based EGMs was reduced by 43% [34]. During 2020 and 2021, the gambling monopoly Veikkaus also closed about 15 gambling arcades across the country. In 2019, Veikkaus had already ended the provision of table games in restaurants and bars.

In addition, the monopoly operator Veikkaus has anticipated forthcoming changes in the Finnish Lotteries Act and introduced mandatory identification in land-based non-casino EGMs in January 2021 and in land-based arcade EGMs in June 2021. Following identification measures, mandatory precommitment in the form of loss limits was extended to EGMs in September 2021 (see Table 1 below for a full list of restrictions). The mandatory identification and precommitment will be extended to all land-based lottery and betting products in 2023.

**Table 1:** Main restrictions on gambling during Covid-19 in Finland

Restriction	Time period
Reduction of land-based EGM numbers and arcades (from 18 500 to less than 10 000 EGMs, closure of 14 arcades during 2022)	2020–2022
**Covid-19 restrictions on EGM and table game availability**	
First wave	14.3.2020–15.7.2020
Second wave*	26.11.2020–6.5.2021
Third wave*	6.8.2021–1.10.2021
Reduced monthly loss limits for fast-paced online gambling (from 2000 to 500 euros)^a*,b*^	1.5.2020–1.10.2020
Reduced daily loss limits for fast-paced online gambling (from 1000 to 500 euros)^a*^	1.5.2020 (permanent change)
Covid-19-related cancellations of sports events**	13.3.2020–15.6.2020
Mandatory identification (non-casino EGMs)^c*^	12.1.2021
Mandatory identification and daily purchase limit (land-based sports betting)	10.6.2021
Mandatory identification (arcade EGMs and table games)	1.7.2021
Mandatory precommitment (all land-based EGMs)^d*^	1.9.2021

* Depending on the hospital district

** Important variations across leagues, dates coincide with the most common restriction periods

**Fig. 1:**
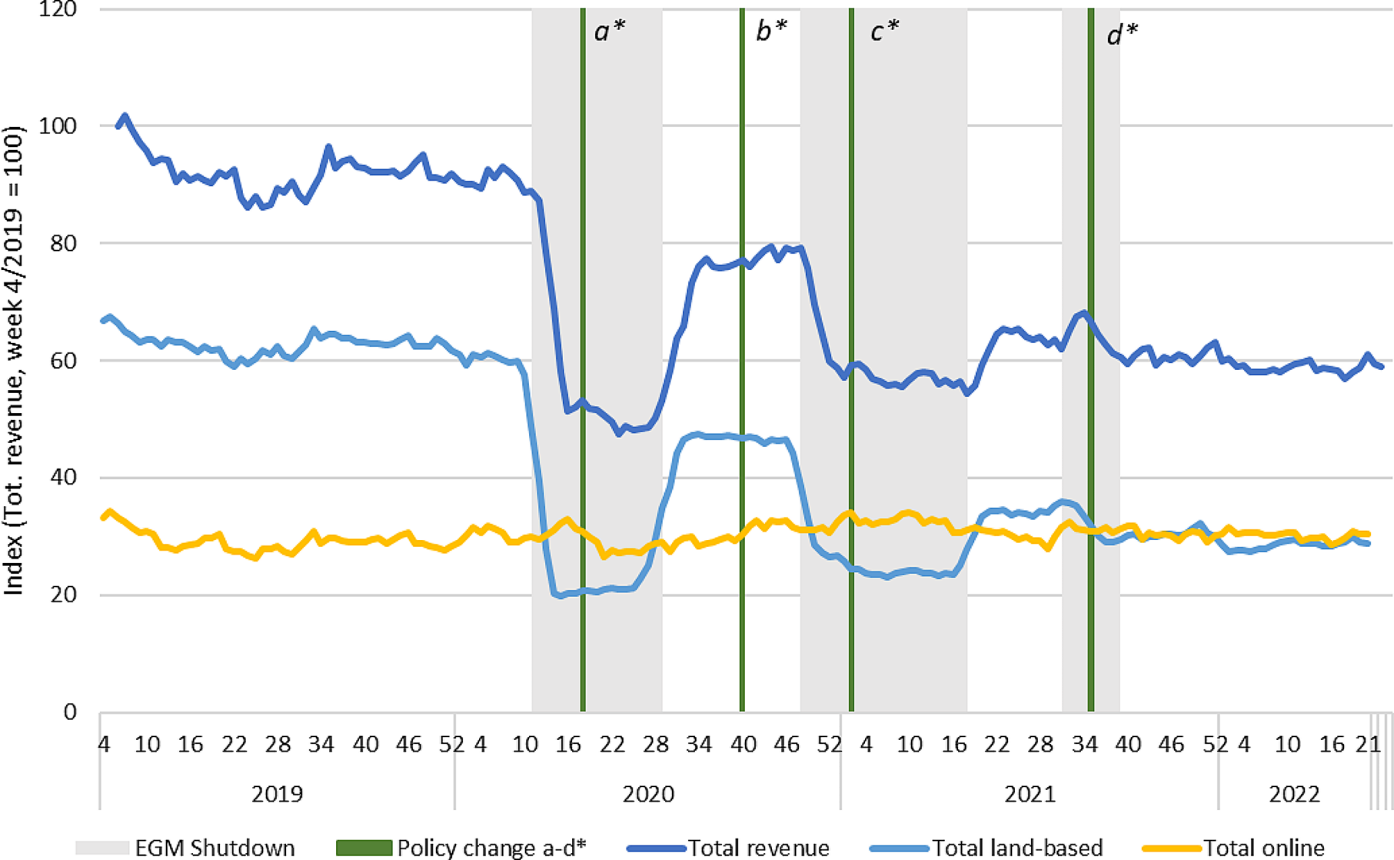
Overall sales of land-based and online gambling in Finland 2019–2022. Total index of revenue and land-based index and online index in relation to total revenue at 4/2019 (Total index = land-based index + online index). Indexed four-week moving averages with week 4/2019 = 100. Policy changes: a) Reduced loss limits for fast-paced online gambling (daily and monthly); b) Return of higher loss limits for fast paced online gambling (monthly); c) Mandatory identification (non-casino EGMs); d) Mandatory precommitment (all land-based EGMs)

**Fig. 2:**
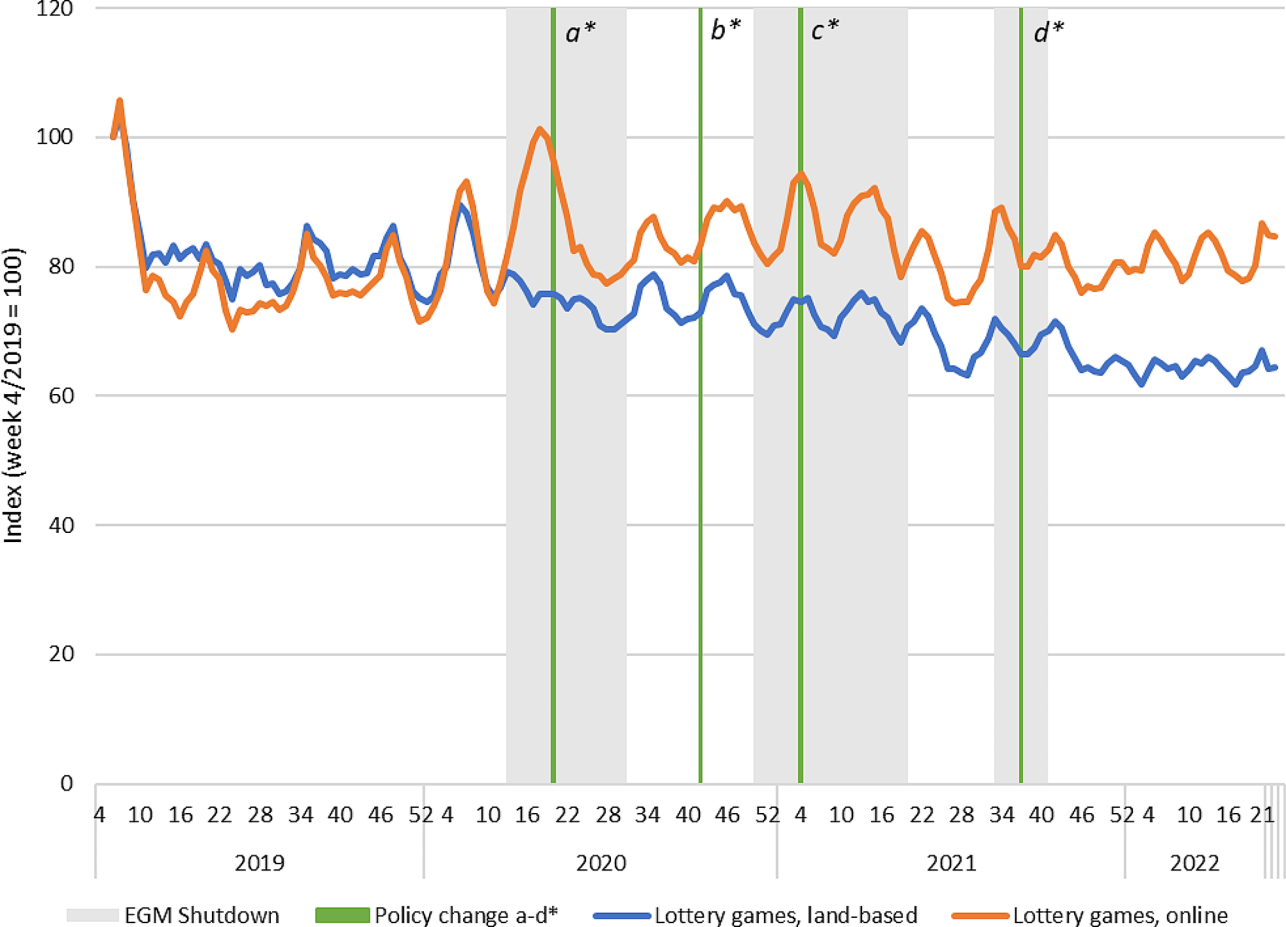
Revenue from lottery-type products in land-based and online channels in Finland 2019–2022. Indexed four-week moving averages with week 4/2019 = 100. Policy changes: a) Reduced loss limits for fast-paced online gambling (daily and monthly); b) Return of higher loss limits for fast paced online gambling (monthly); c) Mandatory identification (non-casino EGMs); d) Mandatory precommitment (all land-based EGMs)

**Fig. 3:**
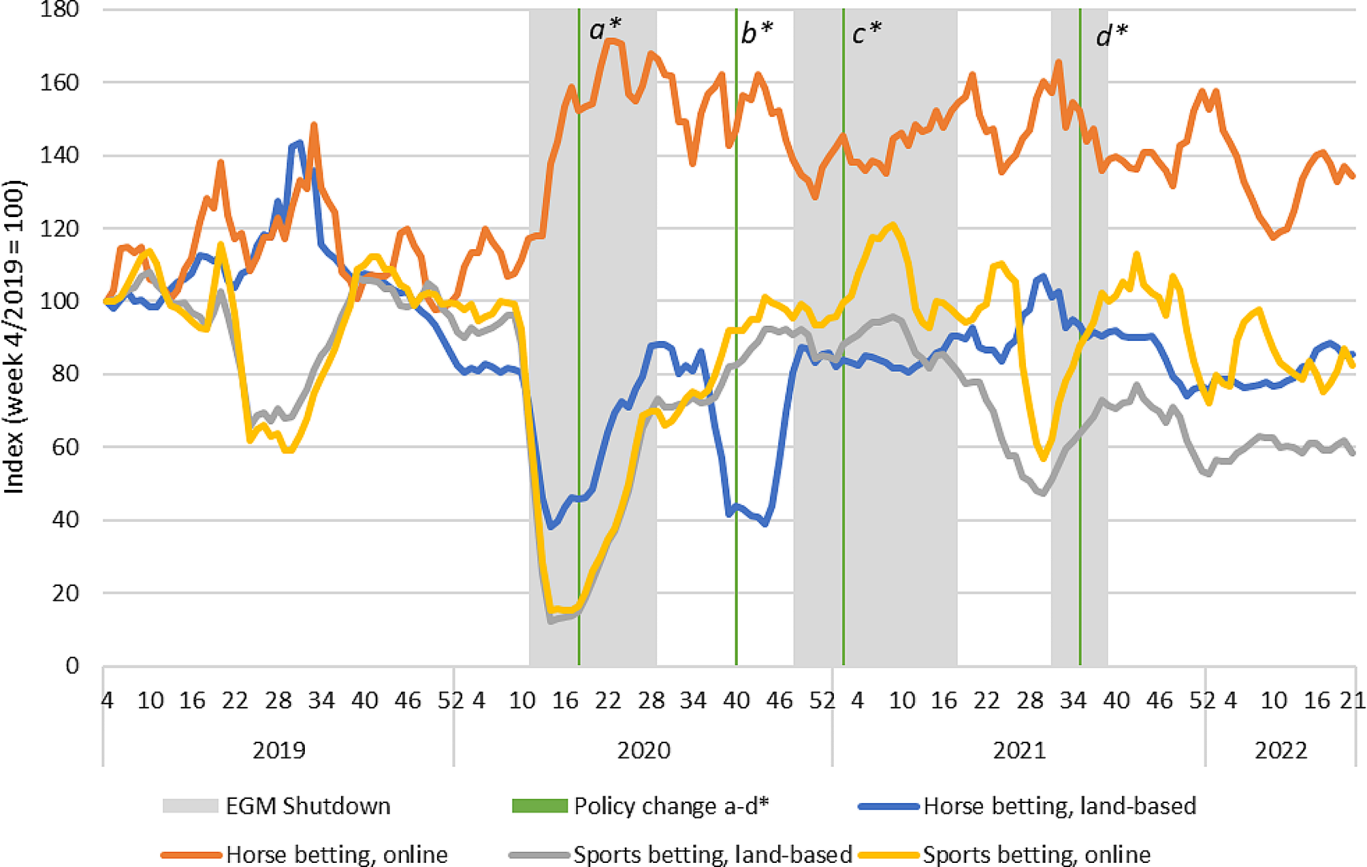
Total revenues of sports and horse betting products in Finland 2019–2022. Indexed four-week moving averages with week 4/2019 = 100. Policy changes: a) Reduced loss limits for fast-paced online gambling (daily and monthly); b) Return of higher loss limits for fast paced online gambling (monthly); c) Mandatory identification (non-casino EGMs); d) Mandatory precommitment (all land-based EGMs).

**Fig. 4:**
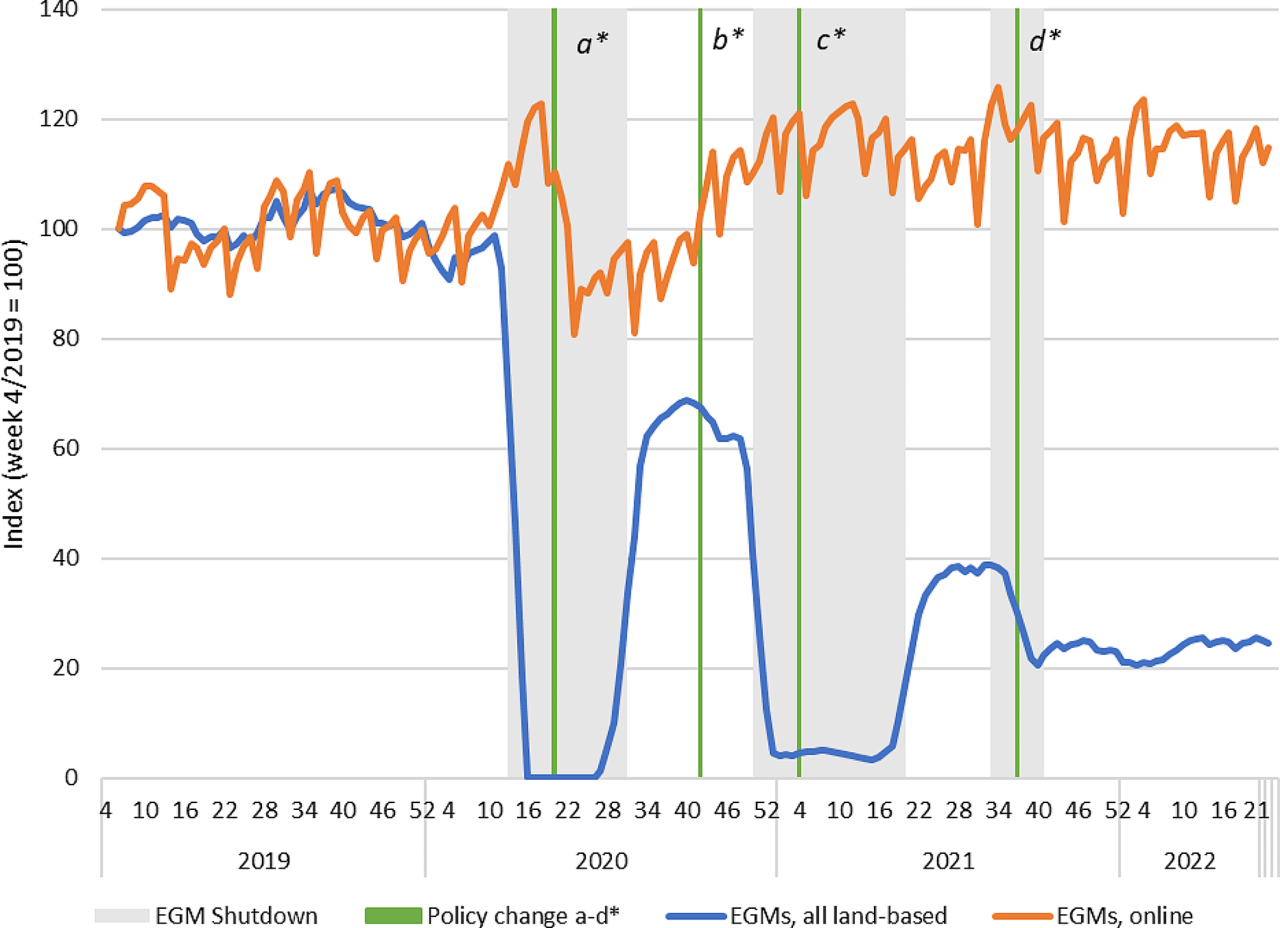
Revenue of EGM products in land-based and online channels in Finland 2019–2022. Indexed four-week moving averages with week 4/2019 = 100. Policy changes: a) Reduced loss limits for fast-paced online gambling (daily and monthly); b) Return of higher loss limits for fast paced online gambling (monthly); c) Mandatory identification (non-casino EGMs); d) Mandatory precommitment (all land-based EGMs)

**Fig. 5:**
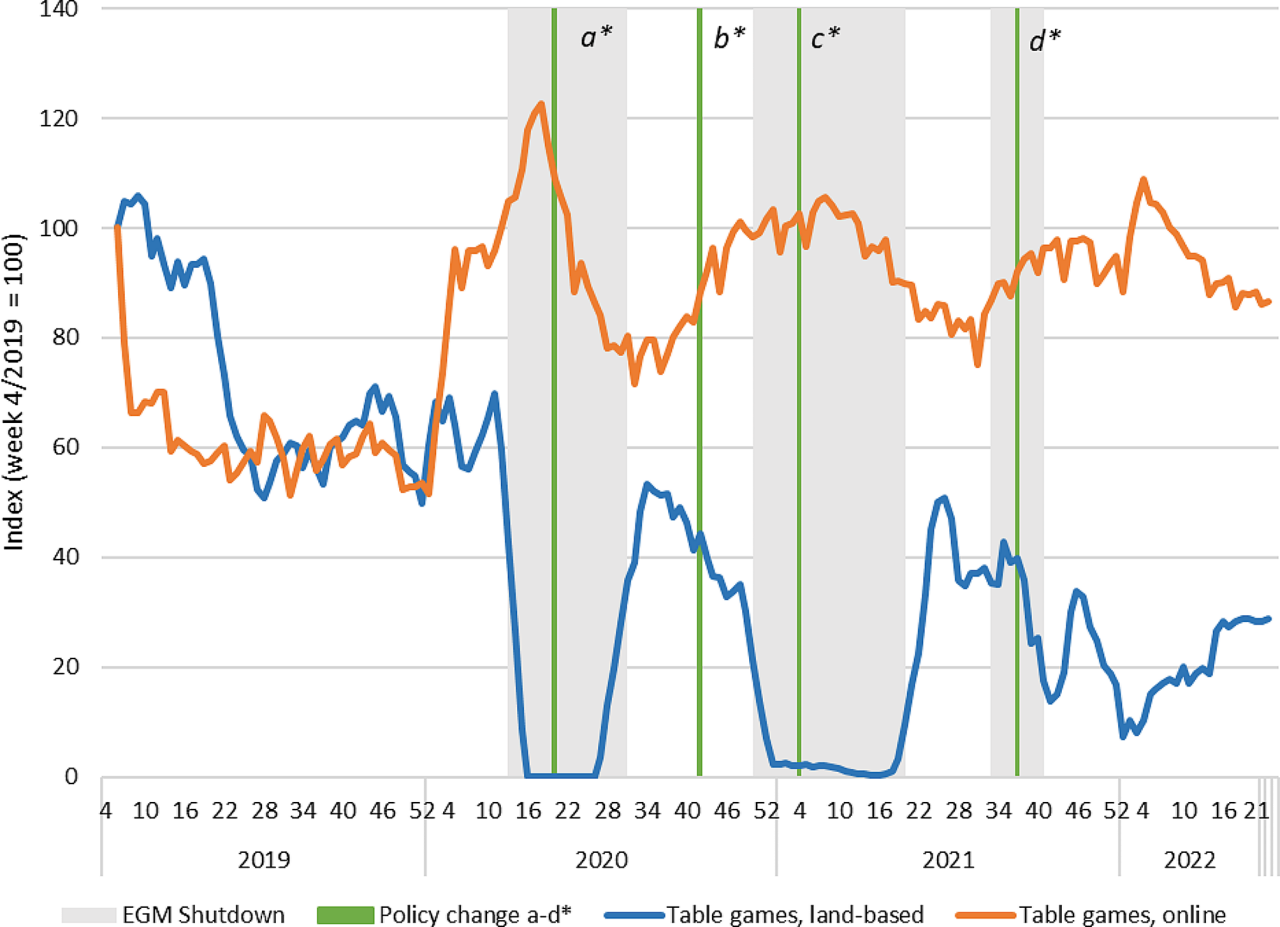
Revenue of table game products in land-based and online channels in Finland 2019–2022. Indexed four-week moving averages with week 4/2019 = 100. Policy changes: a) Reduced loss limits for fast-paced online gambling (daily and monthly); b) Return of higher loss limits for fast paced online gambling (monthly); c) Mandatory identification (non-casino EGMs); d) Mandatory precommitment (all land-based EGMs)

**Fig. 6:**
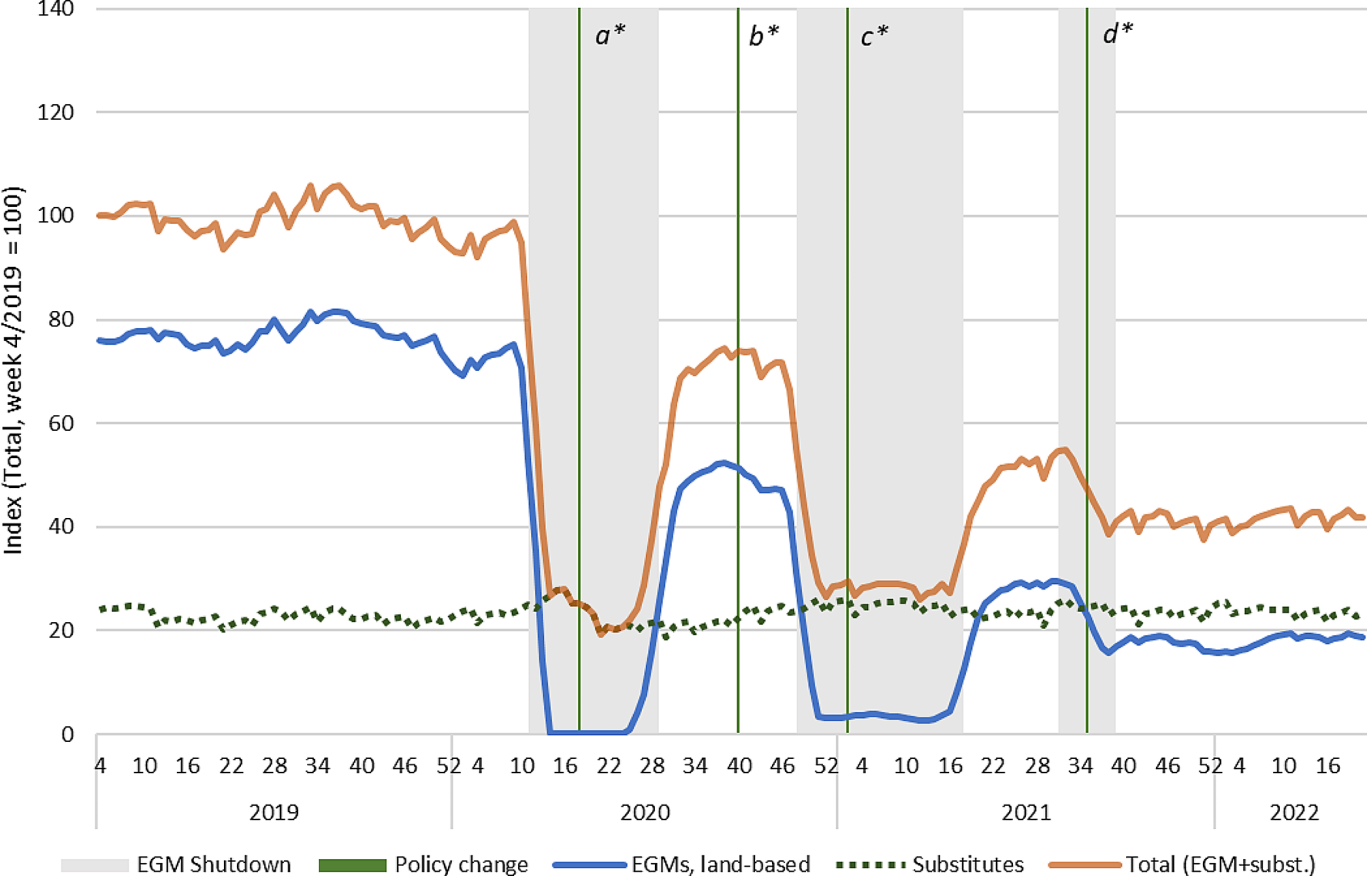
Sales of land-based EGMs and fast-paced substitutes in Finland 2019–2022. Indexed four-week moving averages with week 4/2019 = 100 (Total index = land-based EGM index + online substitutes index, including online EGMs). Policy changes: a) Reduced loss limits for fast-paced online gambling (daily and monthly); b) Return of higher loss limits for fast paced online gambling (monthly); c) Mandatory identification (non-casino EGMs); d) Mandatory precommitment (all land-based EGMs)

^a−d*^ Policy changes shown in Figs. 1, 2, 3, 4, 5 and 6

### Data

The Finnish gambling monopoly Veikkaus has a statutory obligation to provide data to the Finnish Institute for Health and Welfare for research on gambling harms (Finnish Lotteries Act § 55). On the basis of this legal provision, we requested information on weekly gross gambling revenue (GGR) per gambling product type from the beginning of the second week of 2019 (January 7th, 2019) until the end of the 26th week of 2022 (July 3rd, 2022). Veikkaus delivered the data as theoretical loss in euros that we converted to indexes. The theoretical loss measure is often used instead of GGR because it gives a better description of the volume of gambling by disregarding the impact of large wins and by taking into account the role of chance and risk. The gambling industry uses the gross gambling revenue (GGR) and theoretical loss as synonyms, and they are in fact very close each other in the long term [35]. However, because it does not include short-run deviations, the use of theoretical loss instead of GGR in this paper provides a better picture of gambling volume and changes in it.

The gambling product types separated in the categorised dataset included *lottery-type products*, consisting of weekly, bi-weekly and daily draw lotteries (separated between online and land-based sales) and scratch cards (online / land-based sales); *betting-type products* consisting of sports betting (excluding horse racing) (online / land-based sales) and horse race betting (online / land-based sales); as well as *casino-type products* consisting of EGMs (online / land-based sales divided into sales in casinos or arcades and sales in non-casino environments), table games (online / land-based sales), and bingo (online sales only as Veikkaus does not operate land-based bingo).

We also requested Veikkaus for their data on all Covid-19-related restrictions on their operations between March 2020 and end of June 2022. Restrictions referred to any limitations on or closures of gambling offer, casino, arcades, or EGMs, including dates of these restrictions. We also included information on restrictions on online gambling: from May 2020, the loss limit in fast-paced online gambling products was reduced by the Ministry of the Interior to 500 euros a month (from 2000 euros) and 500 euros a week (from 1000 euros). The reduction of the daily loss limit turned out to be permanent, but the reduced monthly loss limit was revoked in October 2020. The same loss limits were applied to land-based EGMs outside casinos in September 2021. In addition, we scanned online resources such as websites of major sports leagues (European and Finnish football and ice hockey leagues) to identify when cancellations of sporting events impacted the selection of betting games. These Covid-19-related restrictions as well as other public health policy interventions into gambling during the time period are described in Table 1.

### Methods

We analysed the effects of Covid-19-related availability restrictions as well as other simultaneously implemented public health policies on the consumption of different gambling products in land-based and online environments, as well as possible substitution effects, using descriptive time series and regression analysis. We were not able to analyse substitution effects at an individual level but at a population level. While not optimal, this type of approach using population-level sales data has also been commonly used in previous research on substitution effects across gambling products [10].

We applied a four-week moving average to the sales data to smoothen the weekly patterns of variation. The first data point was at week 2 of 2019 and thus the first value for a moving average was computed for week 4 of 2019. The figures were further indexed so that week 4 of 2019 equals 100. Index *i* for the moving average *ma* of revenues *rev* for week *w* was computed as

$$\begin{aligned} i_{w,ma} &= 100*((rev_{w - 2}+ rev_{w - 1}\\& \quad +rev_w+rev_{w+1})/4)/rev_{w0}),\end{aligned}$$

where *w*_*0*_ is week 4 of 2019.

Linear regression model for the total theoretical loss as a dependent variable was fitted to the data. Selected gambling restrictions were used as binary explanatory variables. Statistically non-significant (*p* > 0.05) were removed from the final model. The regression coefficients for different effects can be interpreted directly as the change of index points. Possible autocorrelation was considered by computing heteroskedasticity and autocorrelation-consistent (HAC) estimators of the variance-covariance matrix and adjusting the regression estimates and p-values with it.

We compared the sales of land-based products to online equivalents at product category level. The main restriction periods as described in Table 1, are included in the models. In addition, we compared the total sales of land-based EGMs to a sum variable consisting of all other fast paced products that could function as substitutes of land-based EGMs. These substitutes included were scratch cards (online and land-based), online bingo, and online EGMs.

## Results

Overall, there was a marked decrease in total revenue from gambling during the overall period (January 2019 – July 2022). The most important decreases coincide with the restrictions on land-based gambling during the first and second waves of Covid-19. Despite increases in revenue following the lifting of restrictions, the overall consumption does not fully recover, and the decreasing trend follows through the entire period of observation (see Fig. 1).

Linear regression was fitted for the total revenue index. Altogether five statistically significant events were left in the model. The regression estimates for these gambling restrictions showed statistically significant decrease between 9.2 and 15.0 index points of total revenue (see Table 2).

**Table 2:** Linear regression models for the revenue index

	Estimated index points	p
Model constant	90.3	< 0.001***
EGM Shutdown	-15.0	< 0.001***
Reduced monthly loss limits	-10.0	< 0.01**
Reduced daily loss limits	-11.0	< 0.01**
Mandatory identification, non-casino EGMs	-9.3	< 0.01**
Mandatory precommitment land-based EGMs	-9.2	< 0.01**

In the following, we analyse online and land-based sales of different product categories to identify possible product-level substitution effects following the policy changes and availability restrictions.

### Lottery-type products

The sales revenue from lottery-type products (lotteries, scratch cards) remained quite constant during the observation period (see Fig. 2). There were no important policy changes particularly targeting this product group. Overall, there is an observable increase in the sale of online lotteries during the first Covid-19 EGM shutdown, but no similar increases during the subsequent shutdown periods. Overall, lottery sales have declined since 2019 both online and offline, although online sales have picked up to a higher extent.

### Betting-type products

The revenue from betting-type products (sports betting and horse race betting) showed interesting divergence. Online and land-based sports betting have followed very similar sales patterns until late 2020, after which the index of online sales has gradually grown more than that of land-based sports betting (see Fig. 3). The first wave of Covid-19, including important restrictions on major sports leagues, had an important impact on sports betting during spring 2020. Sports betting is also impacted by seasonal effects, as sales tend to decrease over the summer period.

In terms of horse race betting, a substitution of land-based sales by online sales is more apparent. The first and the second wave of Covid-19 are visible as clear reductions in terms of land-based sales of horse race betting, but also notable increases in online sales. Part of the observable increase in online horse race betting may also be attributed to a substitution of other sports betting. Interestingly, the declining trend of land-based sales appears to have begun already before the first wave of the pandemic, but this trend has been amplified during the Covid-19 restrictions. The longer-term sales trend shows that the substitution of land-based horse race betting by online equivalents has been maintained after the Covid-19 restrictions were lifted. The result indicates that online horse race betting acts as a substitute for land-based horse betting but also for online sports betting more generally during a period of restricted availability.

Most policy changes during the observation period, besides the cancellation of major sporting events in spring 2020, concerned casino-type products. These policy changes do not appear to have had important effects on betting sales, either in terms of reducing or increasing the betting product revenue. However, Veikkaus introduced mandatory identification and a daily purchase limit of 1 500 euros for a popular land-based sports betting product on 1 June 2021. This change was followed by a drop in total sales of sports betting and increase in the sales of horse betting products as the purchase limit and mandatory identification was not applied to land-based horse betting. As the sales of online sports betting also decreased at the same time, the drop in overall sales is likely a result of both seasonal and restriction effects. This lends further support to the finding that horse betting is a substitute for sports betting.

### Casino-type products

Land-based casino-type products have been the most impacted category both in terms of Covid-19 restrictions and other public health policy measures during the observation period. All sales of land-based EGM and table games ended during the first wave of Covid-19, and most sales were halted during the second wave, except for a few hospital districts with a less pressing pandemic situation. Restrictions during the third wave were shorter and less prevalent. In 2021, the mandatory extension of mandatory identification and precommitment measures to land-based gambling concerned only casino-type products.

During the overall period 2019–2022, land-based sales of both table games and EGMs have declined considerably while online sales have been maintained at close to baseline levels. Land-based sales of EGMs and table games are likely impacted not only by Covid-19, but also other reductions in availability consisting of the reduction of non-casino EGMs and permanent closures of arcades during 2020 and 2021. The availability of land-based casino products thus also decreased permanently over the observation period. The sales trends of casino products, separated by EGMs and table games, are described in Figs. 4 and 5.

In addition to horse race betting, table game products are the second category with clear observable substitution of land-based sales by online sales. The initial increase in online sales took place already before the pandemic (second week of January 2020) (Fig. 5). This appears to be a seasonal effect as a similar increase with a subsequent drop appears to have also taken place during early January in 2019 and 2021. The overall revenues of table games are also low in comparison to other product categories, making them more sensitive to the impacts of, for example, major events. In 2020, the seasonal increase was nevertheless followed by another increase following the shutdowns of land-based table games, suggesting a substitution effect.

In terms of sales figures, EGM gambling was by far the most profitable product category for Veikkaus before the pandemic. The overall sales trends presented in Fig. 1 therefore follow the developments in the EGM market (Fig. 4). There was no clear substitution of land-based EGM sales with online alternatives, despite Veikkaus operating same game products in both channels. The time series shows an initial 20-point increase in online EGMs during the first wave of Covid-19, but the levels of sales sank below the baseline already before land-based opportunities were reopened. This is likely due to the lowered loss limits for fast online gambling products implemented in May 2020. During the second wave of restrictions, there is no clear substitution effect. However, there is a discernible increasing trend in the revenue of online EGMs after the lockdown commenced. Sales of land-based EGMs have also not recovered after the first wave of Covid-19 and there is a clear declining trend over the full period.

The mandatory precommitment for land-based EGMs and table games outside casinos in September 2021 are also visible as clear drops in terms of the sales of these products. These drops have not been substituted by equivalent rises in terms of online sales. The slight increase in the sales of online table games is more likely attributable to the seasonality effect.

### Land-based EGMs and fast-paced substitutes

As the product-level analysis did not show any significant substitution of land-based EGMs by online alternatives, we also analysed whether land-based EGM revenue was substituted by other products than online EGMs. While all gambling products can in theory substitute each other, we only included the closest substitute products (other fast-paced or intensive forms of gambling: online EGMs and bingo, as well as online and land-based scratch cards and table games). However, the aggregate substitute category did not show any further substitution effects than what was observed regarding online and land-based EGMs during the observation period (Fig. 6).

This finding suggests that public health-oriented policies such as limited availability and mandatory pre-commitment in one product group can reduce total consumption, and consumption will not necessarily move to alternative products. However, it is important to note that the baseline difference in sales volume of land-based EGMs and the substitutes was significant. Therefore, even observed substitution effects did not fully compensate for the substantial decrease of EGM gambling, and rather translated to a decrease in total gambling losses.

## Discussion

This paper has used longitudinal sales data provided by the Finnish gambling monopoly Veikkaus to investigate total sales and possible substitution effects across gambling products during Covid-19-related availability restrictions and other public health measures that have aimed at limiting consumption. Overall, the results have shown that gambling consumption has declined during 2019–2022. This decline stems mainly from reduced consumption in land-based EGMs. For decades, non-casino EGMs had been the bedrock of Veikkaus sales, with a GGR of nearly 700 million euros in 2019. This declining trend is mainly attributable to Covid-19-related restrictions on EGM products. The finding is in line with previous research on the impacts of Covid-19 on gambling consumption from other contexts [14, 15]. However, the trend was further reinforced by other measures taken to limit EGM consumption during the observation period: In 2020–2021, the number of EGMs and arcades was reduced, and land-based EGM and table game products were included under mandatory identification and precommitment policies. Declines in land-based EGM sales were also not substituted by online alternatives in the long term.

The absence of substitution effects for EGM gambling can be explained by several factors. First, it is likely that EGM products were already starting to lose in popularity prior to the observation period. The reductions of non-casino EGMs in Finland were motivated by negative public opinion on the Finnish gambling monopoly and its strategies that were not considered to be in line with its legal mandate to prevent and reduce gambling harms [34]. Second, it is possible that there may have been more important substitution if availability restrictions had targeted land-based EGMs only, without action on the online channel. There was an observable initial rise in sales of online EGM products in March-April 2020. This rise is likely to have been curbed by the temporary reductions in monthly loss limits (from 2000 euros to 500 euros) for fast-paced online gambling products, introduced in May 2020. Once the monthly loss limit of 2000 euros was readopted in October 2020, the sales of fast-paced online games increased. Third, it is also possible that some substitution has occurred that is not visible in the sales of Veikkaus. Between 2019 and 2021 the share of the non-regulated offshore sites increased from 35 to 41% of the total Finnish online gambling market in terms of GGR (according to the estimation of H2 Gambling Capital). If there has been substitution by offshore provision, this has, nevertheless, been relatively minor: It is estimated that the revenue of offshore online casino games increased by approximately 65 million euros between 2019 and 2021 [36].

We also looked at possible substitution effects across other product categories besides EGMs. The only products that showed a clear substitution of land-based sales by online alternatives were horse race betting and table games. The sales of online table games may have been impacted by a renewed interest in poker during the pandemic. A surge in sales of online poker was observed also in other contexts, including the United Kingdom, France, and the United States during the first lockdown [37]. The accompanying declines in the consumption of land-based table games were due to Covid-19 closures but also other reductions in availability (removal of table games from restaurants and bars in 2019; reductions in the number of arcades in 2020–2021). Horse race betting is also an interesting case. During the first wave of Covid-19 in spring 2020, horse races were not organised in Finland. However, races were run in neighbouring Sweden, and Finnish punters were able to place bets on these. The offer of horse racing in Finland has become to depend increasingly on Swedish offer already prior to the pandemic. Swedish races are sold across the Nordic countries, which was also reflected in high sales figures for the Swedish horse racing company during 2020 [38]. The lack of other sports betting options during spring 2020 is also likely to have translated into some short-term substitution of other sports betting by online horse race betting.

Although land-based gambling has not been substituted by online alternatives in the short term, the results have also shown a discernible increasing trend of online gambling (notably online betting products and table games). In 2019, before the onset of Covid-19, Veikkaus reported that 32% of its sales took place online [32]. In 2021, the company reported that 51% of its revenue stemmed from the digital channel [39]. The digitalisation of the gambling monopoly sales is also accompanied by a gradually growing offshore gambling market [39]. The increase in the digital channel is not necessarily related to Covid-19 or other restrictions on land-based gambling, but rather to a more general trend towards digitalisation in the field of gambling [40]. The digitalisation of gambling brings along new challenges for harm prevention. Effective harm prevention measures, such as reduced availability or accessibility, are not as straightforward to implement in online environments where gambling opportunities are constantly available within and beyond licensed offer [41]. It is crucial for researchers and policymakers to consider how gambling harms can be best prevented and addressed in online and digital environments.

This study has some limitations. First, the study has concerned the context of Finland which has been a peculiar case due to the high prevalence of land-based EGM gambling prior to the pandemic. It is likely that substitution effects across gambling products would be different in other contexts. The study period 2019–2022 was also characterised by several restrictions within the Finnish gambling field, particularly concerning EGMs. This limits the possibility of identifying impacts of individual measures on total consumption. Second, we were only able to analyse data from the gambling monopoly Veikkaus, and not providers operating in the offshore online market. Some substitution effects may therefore not be visible in these data. Third, we were only able to analyse substitution effects at a population level.

Further studies should explore substitution across licensed and unlicensed gambling provision and address substitution at individual levels. Comparative studies would also be needed to identify how different public health-oriented policies impact consumption and harms. We have nevertheless shown that reductions in the availability and accessibility of harmful forms of land-based gambling can be an effective strategy to reduce consumption, and that this consumption is not automatically substituted by online alternatives. Substitution effects occur in some products, but this is not complete.

## Conclusions

This paper has used longitudinal gambling company sales data (2019–2022) from the Finnish gambling monopoly to explore the effects of public health-oriented policies in land-based gambling. The results have shown that availability restrictions and mandatory precommitment have reduced the total consumption of gambling in land-based environments. This reduction has not been offset by increases in online environment nor by other products.

These results have important implications for an effective public health approach to gambling. The results have shown that restrictions targeting the overall availability of and exposure to gambling are effective in reducing total consumption, particularly for harmful forms of gambling such as EGMs (cf. [42–44]). In line with previous results from Norway [7], the results show that even full closures of land-based EGMs do not translate into important increases in the sales of online alternatives, at least in the short term. Furthermore, mandatory loss limits on both online and land-based gambling can further reduce total consumption, most probably including harmful consumption. From a public health perspective, these results suggest that a full-population approach, even for single harmful product categories, is effective in reducing consumption and subsequent harms (cf. [5]). 

We conclude that, overall, public health policies such as availability restrictions and mandatory precommitment can be effective public health strategies to tackle the wide range of harms that gambling causes for individuals, families, and societies. Effective policies are also needed to address the harms in the growing digital gambling markets.

## Data Availability

The data that support the findings of this study are available from the Finnish gambling monopoly Veikkaus but restrictions apply to the availability of these data. The data were used under license for the current study, and so are not publicly available. Data are however available from the authors upon reasonable request and with permission of Veikkaus. Please contact the corresponding author (virve.marionneau@helsinki.fi).

## Abbreviations

Covid-19: Coronavirus disease 2019
EGM: Electronic gambling machine
GGR: Gross gambling revenue
